# The Depletion of Nuclear Glutathione Impairs Cell Proliferation in 3t3 Fibroblasts

**DOI:** 10.1371/journal.pone.0006413

**Published:** 2009-07-29

**Authors:** Jelena Markovic, Nancy J. Mora, Ana M. Broseta, Amparo Gimeno, Noelia de-la-Concepción, José Viña, Federico V. Pallardó

**Affiliations:** 1 Department of Physiology, Faculty of Medicine, University of Valencia, Valencia, Spain; 2 CIBERER (Centro de Investigación Biomédica en Red de Enfermedades Raras), Valencia, Spain; 3 Core Research Facility, Faculty of Medicine, University of Valencia, Valencia, Spain; Universidade de Brasília, Brazil

## Abstract

**Background:**

Glutathione is considered essential for survival in mammalian cells and yeast but not in prokaryotic cells. The presence of a nuclear pool of glutathione has been demonstrated but its role in cellular proliferation and differentiation is still a matter of debate.

**Principal Findings:**

We have studied proliferation of 3T3 fibroblasts for a period of 5 days. Cells were treated with two well known depleting agents, diethyl maleate (DEM) and buthionine sulfoximine (BSO), and the cellular and nuclear glutathione levels were assessed by analytical and confocal microscopic techniques, respectively. Both agents decreased total cellular glutathione although depletion by BSO was more sustained. However, the nuclear glutathione pool resisted depletion by BSO but not with DEM. Interestingly, cell proliferation was impaired by DEM, but not by BSO. Treating the cells simultaneously with DEM and with glutathione ethyl ester to restore intracellular GSH levels completely prevented the effects of DEM on cell proliferation.

**Conclusions:**

Our results demonstrate the importance of nuclear glutathione in the control of cell proliferation in 3T3 fibroblasts and suggest that a reduced nuclear environment is necessary for cells to progress in the cell cycle.

## Introduction

Oxidative stress is able to modulate cell growth [1], [2] and it was traditionally defined as the prevalence of the reactive oxygen species (ROS) over antioxidants [3]. Nowadays, a new definition is proposed: disruption of redox signalling and control [4]. This takes into account the signalling role of ROS that emerges in many physiological processes, including cell proliferation. Classical reports by Oberley and Davies showed that mild extrinsic oxidative stimuli, such as superoxide and hydrogen peroxide, could activate signalling pathways leading to proliferation [1], [5]. Subsequently, it was shown that a low level of ROS is necessary for the correct mitogenic signalling [6]. The later finding that an oxidation event early in G1 phase is a critical regulatory step in the progression to S phase, lead to the development of the model of redox cycle within the cell cycle; the transient change in ROS could modify the redox state of cell regulatory proteins at their critical cysteine residues and thus determine the progression or arrest in the proliferation [7], [8].

However, little information has been provided on the active role of glutathione and other powerful antioxidant cellular defense mechanisms during the cell cycle.

A number of seminal previous reports must be considered. Early studies suggested the role of low molecular weight thiols in the cell proliferation [9], and point to the level of GSH as an important factor in the control of the tumour growth [10]. Nevertheless, it was only relatively recently when the group of Dean Jones defining the cellular redox environment by estimation of the ratio of glutathione/glutathione disulfide couple concluded that each phase in the life of the cell is characterized by a particular redox state and that proliferating cells are in a most reduced state [11]. In general, the elucidation of the role of GSH in the cell proliferation was approached by the determination of its overall cellular content, although it is the nucleus where most cell cycle progression events take place.

The study of nuclear compartmentalization of GSH poses a significant methodological challenge and the findings were controversial over the last decade. Pioneer work by Bellomo et al. [12] using monochlorobimane-GSH conjugation demonstrated an important nuclear compartmentalization of GSH in hepatocytes. However, a report by Briviva et al. [13] on microinjection studies performed with various fluorochromes, including monochlorobimane, showed that GSH conjugates can preferentially localize to nuclei [13]. In addition, nuclear pores do not restrict diffusion of low molecular weight solutes like GSH [14], which could possibly difficult the establishment of a specific pool of GSH within the nucleus. Several studies have shown that glutathione related enzymes like GSH S-transferase (GST) and GSSG reductase isoforms are not uniformly distributed in nuclei and cytoplasm [15]. For instance of the 8 reported isoforms of GST two of them have been found in the nucleus according to different authors [16], [17]. Although other report denied the presence of any GST activity in the nucleus [18] various reports [19], [20] including a recent work by Stella *et al.* [21] clearly showed that at least α-GST isoform is present, both inside the nucleus and in the outer nuclear membrane, suggesting that GST plays a major protective role in the nucleus against alkylating compounds and organic peroxides. Apart from glutathione, another physiological reducing agent in the nucleus, thioredoxin-1 and other related systems also redistribute between nuclei and cytoplasm creating a protective reduced environment within the nucleus. A recent report by Gutscher *et al.* [22] provides a new and valuable tool to study the glutathione redox state in cells. Although the method is restricted to cytosol and mitochondrial compartments, this kind of methodology can provide a better insight in the redox state of the different cellular compartments during cell proliferation.

In a series of previous reports we showed that cells have high GSH levels when they proliferate, and cellular GSH levels correlate positively with telomerase activity [23], [24]. When we characterized the distribution of GSH within the cell [24], a nucleus:cytoplasm (N/C) ratio higher of 4/1 was shown in 3T3 fibroblasts during the early phases of cell proliferation. The N/C ratio decreased to 1/1 when cells reached confluence, i.e. when most cells were in the G0/G1 phase of the cell cycle, suggesting that cells need a reduced nuclear environment to proliferate.

GSH concentration can be selectively decreased *in vivo* by various methods, e.g. by buthionine sulfoximine (BSO) which is a transition state inactivator of glutamate cysteine ligase (GCL) that catalyzes the first limiting step of GSH synthesis. Alternatively, it can be inhibited by non-specific agents: diamide (a thiol-oxidizing agent), N-ethylmaleimide (a thiol-alkylating compound) and butylhydroperoxide [25]. Diethylmaleate (DEM) also decreases intracellular GSH concentration through a reaction catalyzed by the enzyme glutathione-S-transferase [26].

The aim of this work was to clarify the importance of nuclear GSH in cell proliferation. Cells were treated with BSO or DEM. These agents decrease GSH levels by two different means; BSO decreases GSH synthesis and DEM, a weak electrophile forms DEM-GSH adducts, although DEM may not be absolutely specific to GSH [27]. We found that DEM but not BSO decreased nuclear GSH and impaired cell proliferation. We show for the first time that cellular proliferation specifically relates with nuclear, but not with total cellular GSH levels. The results underscore the importance of maintaining a reduced nuclear environment in order to maintain normal cell cycle progression.

## Materials and Methods

### Cell Culture

3T3 fibroblasts were cultured in Dulbecco's modified Eagle's medium supplemented with 10% foetal calf serum (Invitrogen, San Diego, CA, USA) and antibiotics (25 U/ml penicillin, 25 µg/ml streptomycin, and 0.3 µg/ml amphotericin B) (Invitrogen, San Diego, CA, USA) in 5% CO_2_ in air at 37°C in 25 or 75 cm^2^ flasks.

### Glutathione depletion

3T3 fibroblasts were plated as previously described [24] and after attaching to the plate (3 h) GSH levels were manipulated by one of the following mechanisms:

- Incubation with 100 µM diethylmaleate (DEM) (Sigma-Aldrich, St. Louis, MO, USA);
- Incubation with 100 µM DEM and 1 mM glutathione ethyl ester (GSHe) (Sigma-Aldrich, St. Louis, MO, USA);
- Incubation with 10 µM Butionine sulfoximine (BSO) (Sigma-Aldrich, St. Louis, MO, USA)

### Enzymatic determination of reduced glutathione

Cultured fibroblasts were washed with PBS and extracts were obtained in 6% perchloric acid (PCA) containing 1 mM EDTA. GSH was measured spectrophotometrically using the glutathione-S-transferase assay [28].

### Cell cycle Analysis by Flow Cytometry

Cell cycle phases were determined by the estimation of the DNA content using propidium iodide (PI) staining and flow cytometry analysis [29], [30].

Fixation: Cells were collected by trypsinization and counted. The pellet was fixed in ice cold 96% ethanol (1×10^6^ cells/2 ml ethanol). Samples were stored at −20°C until analysis.

Staining: Ethanol fixed cells were washed twice with PBS at room temperature and incubated for 1 h at room temperature in the staining solution that contained: 50 µl propidium iodide (1 mg/ml) (Sigma-Aldrich, St. Louis, MO, USA), 50 µl IGEPAL (1∶10) (Sigma-Aldrich, St. Louis, MO, USA) 25 µl RNAse (10 mg/ml) (Sigma-Aldrich, St. Louis, MO, USA) and 875 µl of PBS per 1×10^6^ cells.

Analysis was performed using an EPICS ELITE cell sorter (Coulter Electronics, Miami, FL, USA). Propidium iodide was excited with an argon laser tuned at 488 nm. Forward-angle and right-angle light scattering were measured. Samples were acquired for 15,000 individual cells. Cell cycle phases and cell death were determined at 630 nm fluorescence emission [24].

### Confocal microscopy

Confocal images were acquired using a Leica TCS-SP2 confocal laser scanning unit equipped with argon and helium-neon laser beams and attached to a Leica DM1RB inverted microscope (*Leica* Microsystems, Mannheim, Germany).

3T3 fibroblasts were maintained in culture as described previously [24] and plated in 2 cm^2^ LAB-TEK II chambered cover glass (Nunc, Thermo Fischer Scientific, Waltham, MA, USA) for 5 days, 72 h, 48 h, 24 h, and 6 h before the experiment and treated after attaching as explained before. All treatment conditions and controls were dyed and analyzed on the same day. Double staining was performed: 2 µg/ml Hoechst (Sigma-Aldrich, St. Louis, MO, USA) to localize nuclei and 5 µM green 5-chloromethyl-fluorescein diacetate (CMFDA) (Invitrogen, San Diego, CA, USA), to detect GSH (specificity 95%) [31]. Cells were first stained with 5 µM CMFDA in cell culture medium for 30 min., at 37°C and 5% CO2. After washing with pre-warmed cell culture medium, cells were left to rest for 30 min at 37°C and 5% CO_2_, in cell culture medium. In the last 5 min of incubation 2 µg/ml Hoechst (Sigma-Aldrich, St. Louis, MO, USA) was added. After incubation with the fluorochromes, staining solution was replaced with fresh pre-warmed cell culture medium and cells were analyzed. Cell washing procedures did not change glutathione distribution (result not shown).

The excitation wavelengths for fluorochromes were 488 nm for CMFDA, and 364 nm for Hoechst. Fluorescence detection was 510–540 nm for CMFDA, and 380–485 for Hoechst.

### Quantification of the fluorescence emission by area

Perimeters were drawn around the nuclei following the area marked with Hoechst, and the mean of nuclear CMFDA fluorescence was obtained (100 cells per condition in 3 separate experiments). Similarly, the mitochondria area was defined by high perinuclear CMFDA fluorescence, as reported previously [24]. The cellular area was defined by the transmission image and the perimeter that defines cytoplasm was drown around the cell membrane excluding the nuclear area.

### Immunoblot analysis of cell cycle proteins

Equal amounts of protein (10–15 µg) were boiled in sample buffer for 5 min and separated by SDS/PAGE. After electrophoresis, the proteins were transferred to 0.2-µm-pore-size nitrocellulose membrane. Membranes were incubated in blocking solution [5% (w/v) non-fat dry milk in TTBS [25 mM Tris/HCl (pH 7.5)/0.15 M NaCl/0.05% (v/v) Tween 20], for 1 h at room temperature with shaking; blots were incubated with rabbit primary antibody anti-Id2 (c-20) (Santa Cruz Biotechnology, CA, USA), diluted 1∶750 in 1% (w/v) non-fat dry milk TBS-Tween (TTBS) overnight at 4°C, or with α tubulin (Santa Cruz Biotechnology, CA, USA), diluted 1∶1000 in 1% (w/v) non-fat dry milk TTBS overnight at 4°C with shaking. Blots were washed three times with 1% (w/v) non-fat dry milk TTBS and incubated with the secondary antibody (rabbit for Id2 and mouse for α tubulin) diluted in 1% non-fat dry milk TTBS conjugated to horseradish peroxidase (Santa Cruz Biotechnology, CA, USA) for 60 min at room temperature. Finally, blots were washed during 5 minutes three times with TTBS and detection was carried out using ECL Western blotting detection reagent (Amersham, GE HealthcareBio-Sciences AB, Uppsala, Sweden). The intensity of the bands was quantified using an Image J 1.34S (Wayne Rasband, Image J. 1.34, National Institute of Health, USA), and the relative Id2/α tubulin band intensity was calculated.

### Immunoblot analysis of oxidized and glutathionylated proteins

To measure the level of nuclear protein oxidation 10 µg of nuclear lysates were derivatized, and the western blotting was performed according to the recommendation of the manufacturer. The level of nuclear protein oxidation was determined by the Oxy Blot protein Oxidation Detection Kit (Intergen Company, Burlington MA, USA) which detects carbonylated proteins.

To obtain nuclear lysate, cells were collected using cellular lysis buffer (5 mM HEPES pH 8.0, 5 mM KCl, 0.5% v/v IGEPAL) on ice during 15 min. and then centrifuged at 1000 rpm. 5 min at 4°C. The pellets were resuspended in nuclear lysis buffer (50 mM Tris HCl pH 8.1, 10 mM EDTA, 1% w/v SDS). Nuclear lysates were obtained in absence of reducing agents.

To determine oxidized proteins carbonyl groups were derivatized to 2,4-dinitrophenilhydrazone (DNP-hydrazone) by its reaction with 2,4-dinitrophenilhidrazine (DNPH). The derivatized samples were separated by electrophoresis in an acrilamide gel followed by western blotting and immunodetection protocols as described previously.

To determine glutathionylated proteins western blotting of nuclear extract were performed as usual, using 10 µg of nuclear protein. Addition of any reducing agents was avoided. Membrane was blocked in 5% (w/v) non-fat dry milk in TBST for 1 h at room temperature, and probed against the anti-glutathione antibody (Virogen, Grater Boston, MA, USA) at the dilution of 1∶1000 in 1% (w/v) non-fat dry milk TTBS over night at 4°C, and secondary antibody, goat anti-mouse IgG (Calbiochem, San Diego, CA, USA) conjugated to horseradish peroxidase, at 1∶7500 in 1% (w/v) non-fat dry milk for 1 h at room temperature. Detection procedure was performed using Amersham RPN 2106 ECL Western Blotting Detection Reagent (GE HealthcareBio-Sciences AB, Uppsala, Sweden).

### Determination of glutathione S-transferase and glutathione reductase activity

To obtain cellular extract we collected the cells with 25 mM glicil-glicine pH 7.4, 150 mM ClK, 5 mM MgSO_4_, 5 mM EDTA-Na_2_, 10 mM β-mercaptoethanol. Cell were subjected to freeze-thaw cycles (frozen in liquid nitrogen and thawed at 37°C) to obtain the cell lysate. After centrifuging the cells at 13000 rpm, for 15 min. at 4°C, enzyme activities in the supernatant were determined. Glutathione S-transferase activity was measured as described by Habig *et al.* [32]. and glutathione reductase as described by Massey and Williams [33].

### Statistics

Results are expressed as mean±SD. The statistical analysis was performed by the least-significant difference test using an analysis of variance (ANOVA). The null hypothesis is accepted for all numbers of those set in which F is non-significant at the level of p≤0.05.

## Results

### Diethyl maleate impairs cell growth

**Figure 1:**
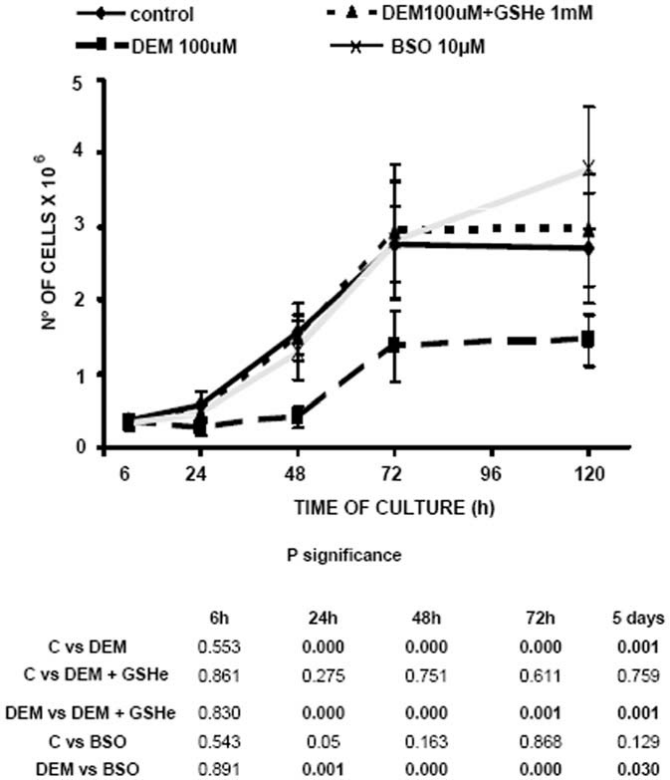
Comparison of the effect of GSH depletion by DEM and BSO on cell growth. Cells were plated and after attaching, treated with 100 µM DEM, or 10 mM BSO, or 100 µM DEM+1 mM GSHe. At 6 h, 24 h, 48 h, 72 h, and 5 days of culture cells were detached by trypsinization and counted. The proliferation curves are created on the basis of the mean±SD of 6–16 different experiments.

Control 3T3 fibroblasts grew slowly during the first 6 hours in culture and then started to grow faster during 48–72 hours. By day 5 of culture cells reached its confluence limit and stopped growing (see figure 1). Fibroblasts incubated with 10 µM BSO exhibited a rate of growth similar to that of controls, even growing for a longer time than untreated cells (day 5 of culture). However cells treated with DEM 100 µM showed a very low growing profile. Five days after plating, less than 1.5×10^6^ cells were present in the culture dish (250,000 cells were plated) compared with more than 2×10^6^ in the untreated group. When DEM-treated cells were co-incubated with 1 mM glutathione ethyl ester (GSHe), to replenish GSH levels, cells grew at a similar rate as controls.

### Depletion of cellular glutathione concentration during cell proliferation

**Figure 2:**
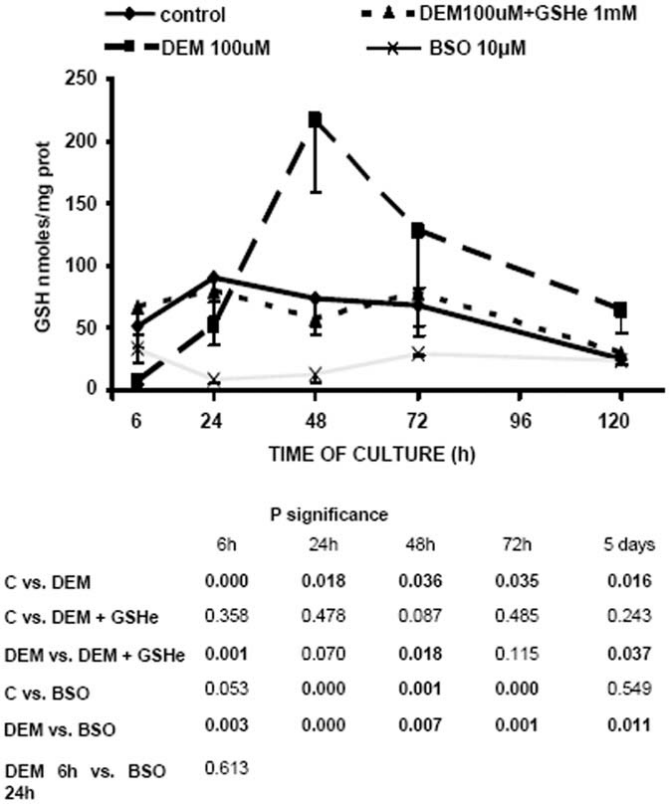
GSH depletion in 3T3 fibroblasts. The total cellular GSH concentration was assessed. The cells were plated as described previously (4) and after attaching 100 µM DEM, or 10 mM BSO, or 100 µM DEM+1 mM GSHe were added. The total cellular concentration of GSH was determined spectrophotometrically as described in “Materials and Methods”. The results are presented as mean±SD of 5–12 different experiments.

Cellular glutathione concentration fell progressively during the culture (as previously reported, in reference [24]). Incubation with DEM caused a marked and immediate decrease in GSH which was followed by a considerable over-shooting at 48 h of culture (see figure 2). Treatment with BSO decreased significantly the GSH level at 24 h of culture, which augmented towards 72 h, but the levels persisted significantly lower than control. However, when cells were incubated with DEM and GSH-ester, glutathione levels were maintained similar to control (no depletion and no over-shooting).

### Changes in nuclear glutathione induced by DEM

We studied the distribution of cellular GSH between cytoplasm and nucleus at different times of culture (6, 24, 48 and 72 hours).

**Figure 3:**
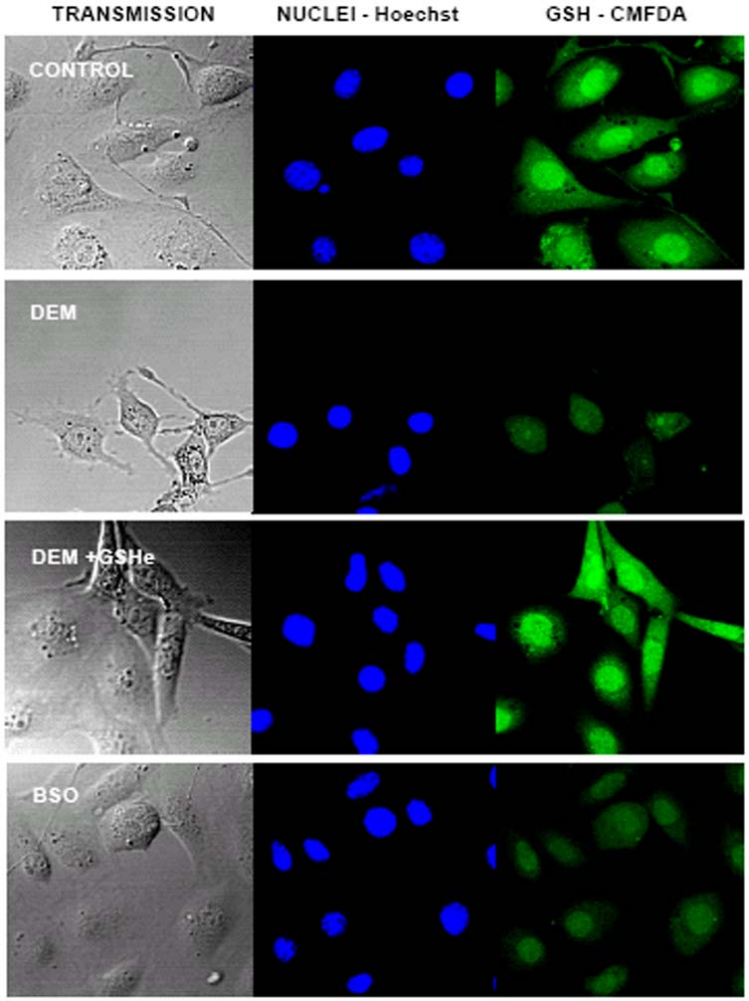
The GSH distribution after its depletion with DEM and BSO. The cells were plated as usual and after attaching, 100 µM DEM, or 10 mM BSO, or 100 µM DEM+1 mM GSHe were added. At 24 h after plating the cells were stained as described in Materials and Methods and observed by confocal microscopy in the chamber provided with 5% CO_2_ and at 37°C. Images were taken by light microscopy (transmission) and by confocal microscopy, as described in Materials and Methods, to capture blue fluorescence of nuclei (Hoechst-nuclei), green fluorescence that marks GSH (CMFDA-GSH) and red fluorescence of dead cells (PI-dead cells) (results not shown). Z series of at least 8 planes were obtained and maximum projection images were created and analysed. The representative experiment (of five) is presented.

Figure 3 depicts untreated fibroblasts during 24 hours in culture showing that GSH (CMFDA staining-green fluorescence) co-localizes with DNA (Hoechst staining-blue fluorescence). However, cells incubated with 100 µM DEM showed a homogeneous GSH distribution within the cells. Surprisingly, inhibition of GSH synthesis by BSO was unable to change the distribution of cellular GSH, showing a cellular glutathione pattern different from the one shown in the DEM-treated cells. Indeed, GSH was high in the nucleus and low in the cytoplasm, like in the control group. Thus, each GSH-depleting agent induces different responses in the distribution of cellular GSH when fibroblasts were proliferating. Co-incubating the cells with 100 µM DEM and 1 mM GSHe showed similar glutathione distribution to untreated cells.

**Figure 4:**
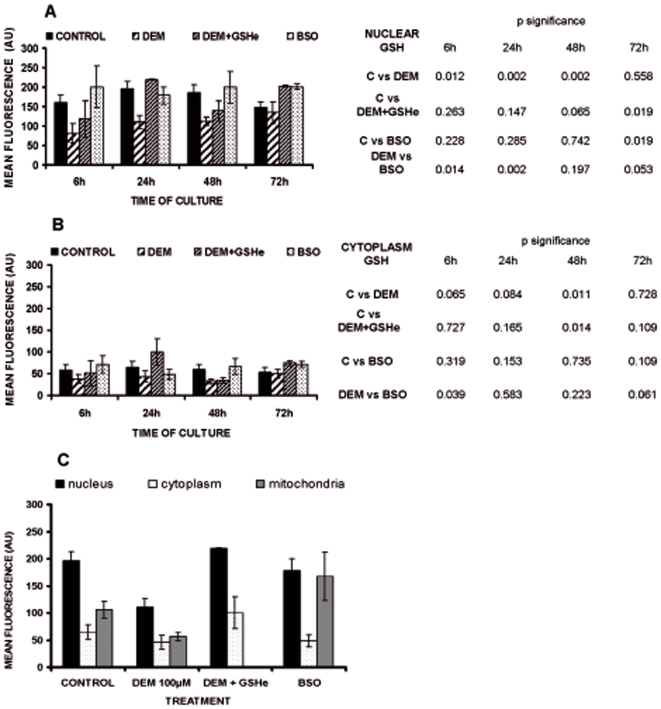
The effect of DEM and BSO treatment on the nuclear and cytoplasmic pool of GSH. The maximum projection images (as presented in the fig. 3) were analysed by area, as described in “Materials and Methods”. CMFDA fluorescence in nuclear area (defined by Hoechst staining, see fig. 3) is presented in fig. 4A and the CMFDA fluorescence of the cytoplasm area (defined by transmission images, see fig. 3) is presented at fig. 4B. The results are mean values of at least 4 different experiments (50–100 cells per experiment). The level of CMFDA fluorescence in the nuclear, cytoplasmic and mitochondrial area after treatment with BSO and DEM at 24 h of culture is presented at the panel C. The analysis of nuclear and cytoplasmic area was performed as described. Mitochondrial area was considered to be marked by perinuclear green fluorescence, as demonstrated previously (4). The results are presented as mean of 3–5 different experiments.

Quantification of the fluorescence emission in the nuclear and in the cytoplasmic area is shown in Figure 4. Panel A shows nuclear CMFDA fluorescence at different time points in untreated, DEM, DEM+GSHe and BSO treated 3T3 fibroblasts. Clearly DEM treated fibroblasts at 6, 24 and 48 hours show lower fluorescence intensity than controls. In those cells incubated with BSO, CMFDA staining was similar or even higher than in control. Replenishment of GSH with glutathione monethyl ester showed nuclear CMFDA distribution similar than untreated cells. Thus, DEM but not BSO is able to maintain low nuclear GSH levels at 6, 24 and 48 hours of incubation.

The mean cytoplasmic CMFDA fluorescence is much lower than in the nucleus due to the extension of its surface. In addition, variability of CMFDA fluorescence is higher due to the variability in the cytoplasmic area. It was shown that both DEM and BSO induced a decrease in cytoplasmic CMFDA fluorescence at 24 hours of culture (figure 4B). No significant changes could be found in the other cell conditions tested; this is probably due to the high CMFDA fluorescence in the perinuclear area, which could be explained by the fact that BSO, at the concentrations used in this work, is unable to deplete mitochondrial GSH [34], [35]. Indeed, the determination of the perinuclear mitochondrial CMFDA staining after co-localization with mitotracker red (as described previously [24]) (figure 4C) in 3T3 fibroblasts at 24 hours of culture show that BSO treated cells maintained a high mitochondrial CMFDA staining but cytosolic fluorescence was indeed decreased by BSO.

### Effect of nuclear GSH depletion on cell growth

**Figure 5:**
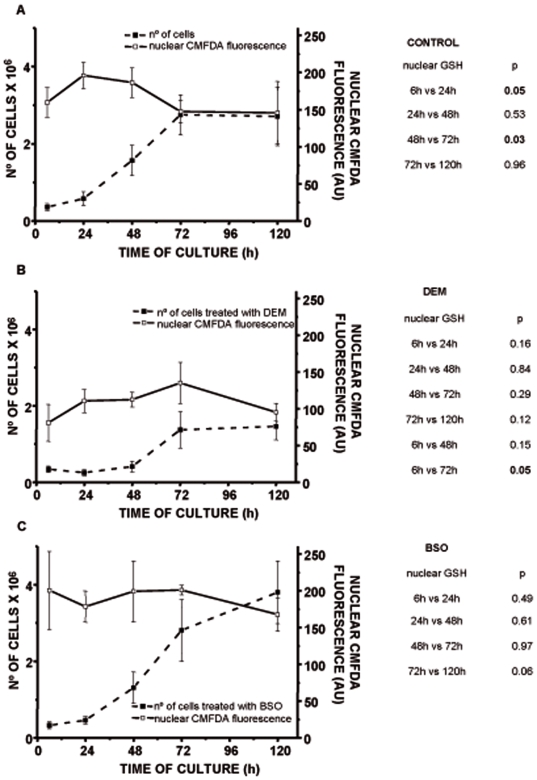
The relationship of the nuclear GSH level and the rhythm of cell growth. The consequences of the GSH depletion by DEM and BSO on the cell growth are shown on the panel B and C, respectively. Control cells are presented at the panel A. The mean CMFDA fluorescence in nuclear area (defined by Hoechst staining, see fig. 3) was obtained as described in materials and methods, and the profile of the cell growth was characterized by cell number. The results are presented as mean values of at least 4 experiments.

Plotting cell growth curves against nuclear CMFDA fluorescence shows than in control fibroblasts nuclear GSH distribution is maximal during cell proliferation (figure 5A). However, in DEM treated cells (figure 5B) nuclear CMFDA fluorescence is lower than in control cells and DEM attenuates and postpones the nuclear GSH peak that takes place during cell proliferation. In BSO treated cells (figure 5C) nuclear CMFDA staining remains high during the time of culture, as high as in untreated fibroblasts, showing a proliferation curve very similar to the one shown in controls.

### Changes in cell cycle induced by DEM but not by BSO

**Figure 6:**
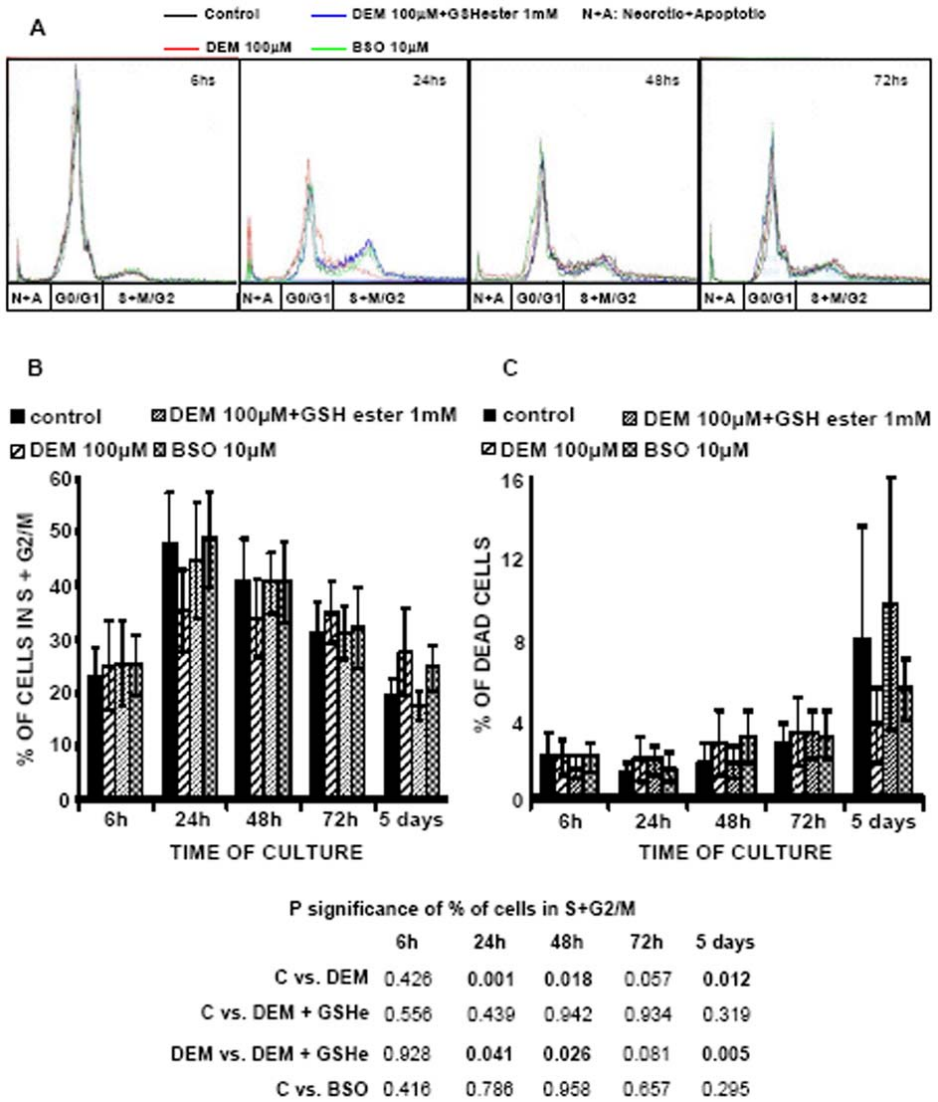
Effect of nuclear GSH depletion by DEM and BSO on cell cycle. Cells were plated and treated as described previously. The cell cycle was studied by flow cytometry, using the level of the fluorescence of the DNA dye propidium iodide (final concentration, 5 µg/mL) at 630 nm fluorescence emission as a measure of the DNA content per cell, as described in materials and methods. The cells were detached, fixed in ethanol and stained along the proliferation curve of the 3T3 fibroblasts. The histograms corresponding to each experimental group at 6 h, 24 h, 48 h, and 5 days of culture (of the same representative experiment) were overlaid and presented at the panel A. The effect of the GSH depletion on the cell proliferation, defined by mean percentages of cells in phases S+M/G2±SD, is presented at the panel B, and the effect of the depletion on the cell death, i.e. the percentage of apoptotic and necrotic cells is shown on the panel C. The results presented are mean±SD of 5–17 different experiments.

Figure 6 (A and B) shows the changes in the percentage of cells in the different phases of the cell cycle during the time of culture. As expected, the percentage of cells in the phases S and G2/M of the cell cycle was higher when cells were growing, i.e. at 24, 48 and 72 hours in culture. But at the beginning of the culture, when cells were plated (6 hours) and when they were confluent (day 5), most cells were in the G0/G1 phase of the cell cycle. However, in cells that were treated with DEM the percentage of cells in either phase S or G2/M was lower when cells were proliferating (24–48 hours of culture) than in controls. As expected BSO and DEM+GSHe treated cells showed results similar to control fibroblasts.

### Changes in cell proliferation are not due to changes in cell death

The different growing behavior in glutathione depleted cells (with DEM) was not due to an increased rate of cell death (figure 6C). Indeed, the percentage of dead cells (necrotic+apoptotic cells according to the DNA content analysis) in DEM and BSO treated cells remained low during the first 3 days in culture. Only during the fifth day of culture i.e. when control and DEM+GSHe treated cells reached confluence, an increase in the number of dead cells occurred, but not in the DEM and BSO treated cells. Thus, as stated above, the difference in the growing profile between the two depleting agents, DEM and BSO, was not due to changes in the number of dead cells.

### Role of nuclear glutathione in the regulation of cell cycle

**Figure 7:**
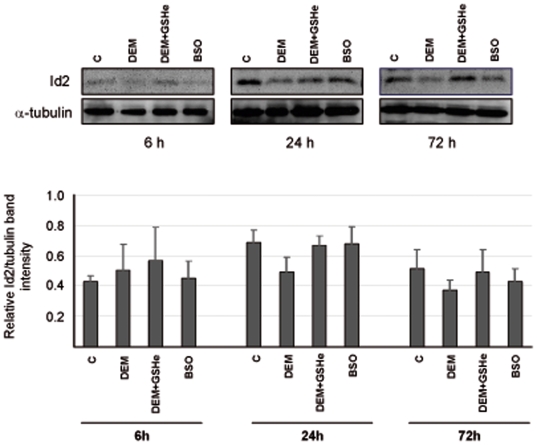
The expression of the cell proliferation marker, Id2, after the depletion of the GSH. The cells were plated as usual and after attaching, 100 µM DEM, or 10 mM BSO, or 100 µM DEM+1 mM GSHe were added. The protein extracts were obtained at 6 h, 24 h, and 72 h of culture and western blotting was performed as described in materials and methods. At upper panel, western blot analysis of Id2 and β-tubulin in 3T3 fibroblasts at 6, 24, and 72 h of culture is shown. Lower panel shows the relative Id2 to β-tubulin band intensity [Mean±SD (n = 4)] derived from densitometry.

In order to find a molecular explanation for the differences in the rate of growth found when glutathione levels were depleted with DEM, we studied the expression of the cell cycle-related protein Id-2 (inhibitor of DNA binding 2). Figure 7 shows that Id-2 expression decreased in the DEM, but not in the BSO treated cells or in the DEM+GSHe treated cells. Thus, depletion of nuclear, but not cytoplasmic glutathione levels is able to decrease Id-2 expression, impairing cell growth.

### Effect of glutathione depletion on Glutathione S-Transferase and Glutathione reductase activities

In view of the major changes induced DEM but not BSO on cell proliferation we determine GST and GR activity in 3T3 fibroblasts (table 1 and table 2, respectively, supplementary data). However both enzymatic activities assayed at two different time points (24 hours and 5 days after plating) showed no differences between control and cells depleted of GSH with either BSO or DEM. Thus the reported changes in nuclear GSH induced by DEM but not by BSO are not caused by variations in the activities of these two important enzymes of glutathione metabolism.

**Table 1:** Effect of glutathione depletion on Glutathione S-Transferase (GST) activity.

Time of culture	Category	GST mU/mg prot.	SD
24 h	control	0,313	0,057
24 h	DEM	0,473	0,077
24 h	BSO	0,398	0,074
6 days	control	0,656	0,047
6 days	DEM	0,715	0,034
6 days	BSO	0,533	0,256

The 3T3 fibroblasts were treated with 100 µM diethylmaleate (DEM) or with 10 µM buthionine sulfoximine (BSO) immediately after attaching. The activity of the GST was determined at 24 h and 6 days of culture, as described in Materials and methods.

**Table 2:** Effect of glutathione depletion on Glutathione reductase (GR) activity.

Time of culture	Category	GR mU/mg prot.	SD
24 h	control	0,071	0,003
24 h	DEM	0,067	0,003
24 h	BSO	0,089	0,000
6 days	control	0,093	0,012
6 days	DEM	0,111	0,003
6 days	BSO	0,112	0,004

The 3T3 fibroblasts were treated with 100 µM diethylmaleate (DEM) or with 10 µM buthionine sulfoximine (BSO) immediately after attaching. The activity of the GR was determined at 24 h and 6 days of culture, as described in Materials and methods.

### Role of nuclear glutathione in proliferating 3T3 fibroblasts; the effect on the protein redox state

**Figure 8:**
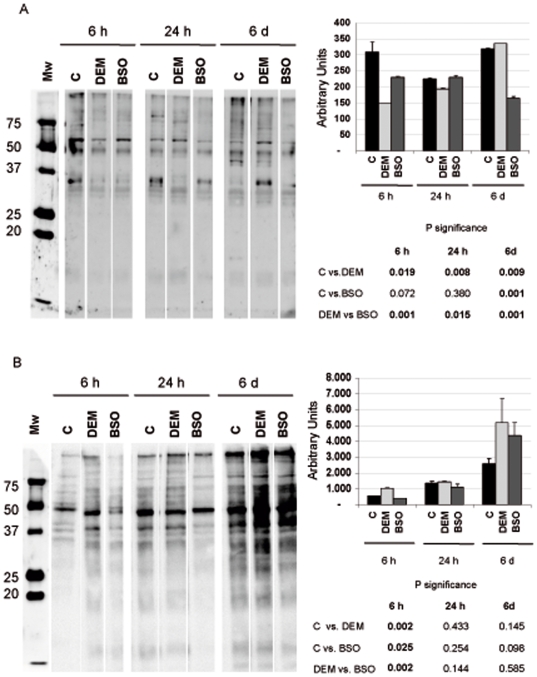
The pattern of overall S-glutathionylated and oxidized nuclear proteins induced by the two GSH depleting agents at 6 h, 24 h and 6 days in culture. A.- Glutathionylated proteins: equal amount of nuclear extracts were loaded and separated by a 12% SDS gel under non-reducing conditions. S-glutathionylated proteins were detected by Western blot using anti-glutathione monoclonal antibody. B.- Oxidized proteins: equal amounts of nuclear extracts were derivatized and separated by electrophoresis in an acrilamide gel. Western blotting and subsequent immunodetection of carbonylated proteins were performed according to the protocol recommended by the manufacturer (see material and methods). Right panels shows the densitometry results for the level of glutathyolation and oxidation of nuclear proteins, respectively [Mean±SD (n = 3)].

In order to study the changes that take part during the short period when GSH is depleted that is not reversed thereafter, we addressed a kinetic assessment of oxidized and glutathionylated nuclear proteins in DEM treated cells versus control. As presented in figure 8 A at 6 and 24 h in culture the fibroblasts showed a sustained decrease in the pattern of nuclear glutathionylated proteins. However, in those cells incubated with DEM that were in culture for 6 days glutathionylation of nuclear proteins increased to values even higher than controls or BSO treated cells. In BSO treated cells the expression of nuclear glutathionylated proteins was similar to controls.

In a similar fashion, the expression of nuclear oxidized proteins was higher when the nuclear GSH was depleted with DEM, with a striking difference at 6 hours of culture, when the nuclear GSH level in these cells was lowest. On the other hand, these cells still have increased oxidized nuclear proteins at day 6 of culture. Thus, protein carbonyl levels did not rebound as glutathionylated proteins. Results show that the depletion of cytosolic GSH with BSO does not affect the redox state of the nucleus, when BSO is used at low concentration (10 µM). However, 100 µM DEM is able to deplete both cytosolic and nuclear glutathione, creating a nuclear environment prone to oxidation.

Clearly, many of the reported effects of DEM on 3T3 fibroblasts last for no more than 3 days and at day 6 DEM treated cells show a profile of glutathionylated proteins similar to controls, although nuclear carbonyls remain higher in cells incubated with DEM.

## Discussion

Cell proliferation is regulated by a variety of mechanisms working to allow the activation and repression of growth stimulatory genes. Transcription factors play an essential role in the regulation of growth control genes. Previous *in vitro* reports show that the activity of these transcription factors is related to its redox environment. In addition, change in the redox potential could induce variations in the activity of those transcription factors. Alterations as small as±15 mV in the redox potential can result in transcription factor translocation and activation or deactivation, depending on the direction of the redox shift [36], [37], [38]. In the present report nuclear oxidized proteins increase when cells are depleted of nuclear glutathione due to DEM action. The change in the nuclear redox environment induced by DEM could activate a shift in the activity of transcription factors that modulate cell cycle as we have demonstrated here with the decreased expression of Id2 in DEM treated cells.

### Discrepancy between total cellular GSH depletion and cell proliferation

Since glutathione is the most important redox regulatory factor [39], a number of reports have focused on the consequences of the depletion of cellular glutathione levels on changes in cellular proliferation [40], [41]. However, all those reports were performed measuring cellular or total glutathione levels, but there is no information relating cellular proliferation with nuclear glutathione levels. A number of studies have indicated the existence of a nuclear GSH pool that resists depletion after exposure of cells to BSO (for a review see [42]. However, Thomas *et al.* [43] showed that depletion of GSH with N-ethyl maleimide or DEM decreased mercury orange fluorescence in the nucleus and cytoplasm to a similar extent. By contrast, mercury orange fluorescence in the nucleus was much more resistant to BSO depletion than that in the cytoplasm. Spyrou and Holmgren [44] showed that inhibition of glutathione synthesis by 0.1 mM BSO was able to decrease GSH synthesis after treatment for 12 hours, but GSH-depleted cells grew as well as control 3T6 cells with no decrease in DNA synthesis. Thus, incubation of cells with low concentration of BSO, although it decreases glutathione levels, does not change cell proliferation. Esposito *et al*. [45] showed that DEM treatment induces cell cycle arrest that is accompanied by several redox-dependent changes in cell-cycle related proteins. Precisely, the p53-independent accumulation of p21 was detected. These authors demonstrated that DEM treatment strongly activates p21 showing a clear inhibition of cell proliferation.

In the present report we provide for the first time information on the comparison of changes in the distribution of GSH in the cell nucleus during cell cycle using the two depleting agents DEM and BSO. Other authors have provided isolated information on the role of BSO or DEM or have described their effect on cell proliferation. However, here we present a comprehensive view, and a possible explanation, for the different proliferative results shown by these two well-known GSH depleting agents

Results presented in Fig. 2 show that inhibition of glutathione synthesis by BSO causes the lasting glutathione decrease strating from 24 h of culture; on the other hand, the effect of DEM is instantaneous with an important rebound effect at 48 and 72 hours. The rebound effect was previously reported by Borroz *et al.* [42] These authors have shown that partial depletion of GSH with either phorone or DEM results in a four- to five-fold increase in hepatic gamma glutamyl cysteinyl synthetase RNA levels.

DEM is an alkylating agent but the effect induced on cell proliferation is not due to its alkylating properties since incubation of DEM and GSHe prevented DEM impairment of cell proliferation.

Here we demonstrate that inhibition of glutathione synthesis by BSO induces a strong decrease in total GSH levels, but the nuclear GSH pool was preserved (Fig. 3. and 4.). Similar results have also been shown by Britten *et al.* [46]. By contrast, depletion of glutathione levels by DEM induces a marked decrease in nuclear glutathione levels. These differences in glutathione depletion compartmentalization could explain the reported differences in the inhibition of cell proliferation, shown in Fig. 1. In fact, our results show that inhibition of glutathione levels by DEM strongly impairs cell proliferation. This difference could be due to the fact that DEM decreases both nuclear and cytosolic glutathione levels in opposition to BSO, which only decreases cytosolic glutathione as it is shown in figure 4C. Indeed, the Fig. 5 represents the relationship between nuclear GSH kinetics and the rhythm of cell growth in the control cells, cells treated with DEM and cells treated with BSO (Fig. 5 A, B and C, respectively). As demonstrated, the depletion of nuclear glutathione severely affects cell growth. Furthermore, the high level of nuclear glutathione is necessary for the exponential phase of the cell growth: these parameters coincide in control cells at 24 and 48 h, and the growth slows at 72 h when the nuclear GSH level is lower; in DEM treated cells the growth is delayed until the higher level of nuclear GSH is reached, which occurs at 48–72 h, while in the BSO treated cells the maintained high level of nuclear GSH between 48 h–72 h results in a prolonged cell growth when compared to control.

We should however point out an apparent contradiction between the results shown using CMFDA and those in figure 2, since the reported decrease in cellular GSH concentration in BSO treated cells, thus not correspond with major changes when the GSH distribution was analyzed by the fluorochrome CMFDA (figure 3,4 and 5). This is due to the fact that the software measures the mean fluorescence value within a designed surface. The size of cells varies during the time of incubation. Cells are small when sided, and then when they grow its cytoplasm spreads in the plate. Later when cells are confluent the size of the cytoplasm shrinks due to the number of neighboring cells. The problem thus not apply to the nucleus since its size remains relatively constant during the time of incubation.

During the last years, the possible nuclear compartmentalization of glutathione related enzymes such as, glutathione S-transferase (GST) isoenzymes has inspired various reports [47], [48], [49], but the precise function (or functions) of the glutathione S-transferases in the cell nucleus remains to be elucidated. Two possible roles have been suggested: biotransformation of electrophilic compounds, protecting the DNA content, where the GST catalyzes the conjugation of glutathione to the xenobiotic compound in a detoxification-related mechanism, and a possible role in modulating gene expression. How GST may contribute to this procedure or which is the exact role of this enzyme in gene regulation is still a question that remains to be answered. However, we could not find significant changes in GST activity when cell were treated with DEM or BSO.

### The role of nuclear GSH: protective and/or regulatory

We show here and in a previous report [24] that nuclear glutathiolation changes during the cell cycle and that the depletion of nuclear GSH changes the pattern of nuclear glutathionylated proteins. The suggestion that reduced nuclear environment could protect oxidant sensitive proteins from oxidation [50] could be confirmed by our results: there was less glutathionilated and more oxidised proteins when the nuclear GSH was depleted by DEM (Fig. 8). However, after nuclear GSH increased (Fig. 4B, 72 h) the glutathionylation of nuclear protiens in DEM treated cells reached the values of control (Fig. 8A), while the level of protein oxidation remained high (Fig. 8B). This reflects the irreversible consequence of nuclear GSH depletion early in the culture and could account for the persisting lower growth rate of DEM treated cells (Fig. 1), despite the important increase in total GSH level. Our study of the expression of Id2 gave support to this assumption; when the nuclear glutathione level is high, the expression of this proliferative marker was also high, while the depletion of nuclear glutathione with DEM caused the important decrease in the level of this protein, and consequently, the decrease in the proliferation. On the contrary, cells treated with BSO maintained their nuclear glutathione level as well as the high expression of Id2 and normal cell growth.

So, the presence of the high glutathione level in the nucleus appears to be a prerequisite for the start of the cell proliferation. Our findings are in line with several other studies aimed to elucidate the fine redox regulatory mechanisms that lie behind the correct cell cycle progression. Conour et al. [50], suggested that the reduction of the intracellular environment as cells progress from G1 to G2/M phase, as shown in our study, may protect genomic DNA from oxidative damage upon brake down of the nuclear envelope. Indeed, one of the assertions in support of this premise derives from the study of oxidative stress related to genotoxicity, recently published by Green [35]; oxidative DNA modifications displayed a negative linear correlation with nuclear GSH. This is of special importance considering the report of Menon et al [7] on the necessity of the oxidative event in early G1 phase to allow G1-S transition. Even more, it has recently been postulated that the restriction of DNA synthesis to the reductive phase of the cycle in yeast may be an evolutionarily important mechanism for reducing oxidative damage to DNA during replication [51], which implies the common mechanism of the DNA protection during S phase in all eukaryotes.

Various studies have demonstrated that the nucleus is more reduced than the cytosol (15 mM GSH vs. 11 mM, respectively) [52], [53], [54]. An important number of nuclear proteins, including transcription factors, require a reduced environment to bind to DNA. More than 62 proteins are involved directly in transcription, nucleotide metabolism, (de)phosphorylation, or (de)ubiquitinylation, which are all essential processes for cell cycle progression [50]. For instance, it appears that, at the onset of cell proliferation in the early G1 phase, an increase of ROS in the cytoplasm is necessary for the initiation of the phosphorylation cascade mediated by epidermal growth factor (EGF) that, subsequently, activates DNA replication and the cell division ([55]). According to Jang and Surh [56] nuclear GSH may act as a transcriptional regulator of NF-*κ*B, AP-1, and p53 by altering their nuclear redox state. The transcription factor NF-κB is an example of distinct redox-sensitive activation and DNA binding [57]; it is activated by various physiological stimuli known to produce ROS; on the contrary, to permit DNA binding, similar to Fos, Jun, and Nrf2, cysteine residue within DNA binding domain must be reduced. Both processes are guaranteed by the adequate redox state of the cytosolic and nuclear environment, respectively. Recently Reddy *et al.* [58] have shown that Nrf2 deficiency leads to oxidative stress and DNA lesions, accompanied by impairment of cell-cycle progression, mainly G(2)/M-phase arrest. Both N-acetylcysteine and glutathione (GSH) supplementation ablated the DNA lesions and DNA damage-response pathways in Nrf2(−/−) cells; however only GSH could rescue the impaired co-localization of mitosis-promoting factors and the growth arrest. In addition, Toledano *et al*. [59] found a redox-dependent shift of oxyR-DNA contacts along genomic DNA, suggesting a mechanism for differential promoter selection.

High level of GSH in the nucleus could provide protection to the proteins against the oxidative threat coming from the cytoplasm at the early phase of cell proliferation [7], and glutathiolation, as it is a reversible modification, could be just the way. On the other hand, based on the simplicity of the redox transition from thiol to disulfide and on the fact that the reversibility was energetically favourable, Cotgrave IA and Gerdes RG [60] more than 10 years ago have proposed glutathionylation as a posttranscriptional modification with the regulative finality. They state that it offers “a strong possibility for transducing “oxidative information” from intracellular oxidants via the GSH redox buffer to individual proteins containing “regulatory thiols”. Also, recently, this posttranslational modification was proposed as a likely molecular mechanism for redox dependent signalling mediated by GSH [61]. Thus, high level of GSH in the nucleus, observed before and at the onset of cell proliferation, could provide the “GSH redox buffer” necessary for the progressing of oxidant stimulated mitogenesis.

On the other hand, as according to interesting hypothesis offered by Bellomo et al. [52] intranuclear accumulation of glutathione may modulate the thiol/disulfide redox status of nuclear proteins and control chromatin compacting and decondensation. Consequently, our finding that the pattern and level of glutathiolation of nuclear proteins change during the cell cycle [24] and with the depletion of nuclear GSH could contribute to the possible implication of this modification in the control of chromatin structure dynamics (for review see [62]).

Since its discovery almost a century ago [63], a number of important functions have been attributed to GSH, however, its outstanding role in nucleated, but not in prokaryotic cells, remains unknown. Our results demonstrate for the first time that it is nuclear GSH levels, and not total cellular glutathione levels, that play a decisive role in cellular proliferation. Our report underscores the important role of nuclear glutathione in cell physiology and suggests that manipulation on nuclear GSH levels could be of paramount importance during development and cancer.
